# Notes and News

**Published:** 1892-01-09

**Authors:** 


### NOTES AND NEWS.

COLLECTED AND COMMUNICATED.

THE Charity Organisation has been called to task. The want of organisation has been laid at its door. Cases of starvation occur in our streets, whilst some of the shelters and refuges contain vacant spaces nightly. The *Globe* offers a practical suggestion that communication in some form or fashion should be held between all such institutions, that an applicant refused for want of room at one shelter might be transferred to some less crowded refuge. This unquestionably would be an advance in organisation, and would, moreover, bring the whole system into closer touch, whereby the actual requirements might be more adequately provided for. The Charity Organisation is an earnest and practically useful institution, and in the particular case through which complaint was raised, it is doubtful, unless a large army of canvassers for applicants for shelter were maintained, whether any amount of systematic management would have averted the evil. Nowhere more than amongst the really poor is pride a living force.

A REPORT by Dr. Felice Santini, a medical officer of the Royal Navy of Italy, addressed to H.E. the Minister of Marine at Rome, gives an interesting account of his mission to the International Medical Congress at Berlin in the autumn of last year. The report deals with a number of important subjects of great practical interest to naval and military surgeons. We may, however, call attention to the observations connected with the subject of naval hospitals in war, their scope, use, allotment, and outfit, and to those on the utilisation of water-carriage of various kinds as a very important auxiliary means of transport in time of war, both in naval and military exhibitions. The prominent part taken by this country is dwelt upon. England, as was only to be expected, has fitted out at different times many excellent hospital ships for the reception and treatment of her wounded soldiers, among which the *Victor Emanuel* may be named as having embodied the largest number of original and practical features. Allusion is made to the splendid success achieved by this country in the prompt and rapid transport of the wounded in Egypt from the field of battle to the land hospitals or the hospital ship by means of boats and steam launches along the Fresh-water Canal and in the subsequent Nile expeditions in that country. There can be no doubt that water transport, wherever it can be used, offers enormous advantages for the removal of wounded and sick men in the way of ease, safety, and comfort; and attempts to develop the system and secure a complete and reliable organisation in this direction are deserving of every consideration in all countries with a seaboard and rivers available.

VOLUMES I. and II. of "Hospitals and Asylums of the World" (J. and A. Churchill) are now ready. They are clearly and concisely arranged for reference. With vol. II. a comprehensive set of plans is published. This part of the work is devoted entirely to matters dealing with Asylum Construction. Volume I. deals with the history and treatment of Insanity in all countries, and all matters appertaining to the inmates of asylums. Volume II. will be found invaluable to architects and those who have aught to do with the erection and arrangement of asylums.

THE American papers have taken up the subject of cigarette-smoking, and they show us some rather remarkable returns. For instance, we are told that two hundred and forty billions of these articles are manufactured during the year, which would give an average consumption of eight thousand a-year by each male American, were they all consumed at home; but then they are not, and as no export returns are given, we cannot fairly shake our heads over immoderate indulgence in the West. In fact, our American cousins regard the habit of cigarette-smoking more seriously than we do. They leave the adult alone—advise him not to inhale, however, and then do not promise him a certainty of consumption, insanity, and certain death, as does the enemy on this side of the water. But what they have done is actually to forbid, by law, cigarette-smoking amongst boys, to whom it is undeniably injurious. Thirty States have adopted the legal course, but in the cities, unfortunately, it has not yet been effective. It is a relief to be assured that cigarettes are no more injurious than pipe smoking, because amongst our female population of the upper classes they undoubtedly hold sway. General taste is undoubtedly against the indulgence on the part of the fair sex, but nevertheless, the habit is greatly on the increase, and one lady informed us not long since that her medical adviser had stated in his opinion he considered the habit, when indulged in moderation, was actually beneficial to women whose nerves were called to bear the strain of the most trying of all things—small daily worries. If this is the case, we advise all husbands who suffer from their wives' overwrought nerves, and these are not a few, to try so simple a remedy. An objection on the score of taste could well be put aside for so important a result of added peace in a household.

BLUE skies and sunshine are inspiriting to well and sick alike, and in this respect we venture to think that the inhabitants of Hastings had very much the best of it this Christmas. The Hastings and East Sussex Hospital basked in the sunshine of a balmy Christmas Eve, and quite tempted one to enter, so bright and pleasant did it look. It is built in Mr. Keith Young's happiest style, and is an ornament rather than otherwise, in its prominent position facing the pier, and overlooking the esplanade and sea. Such provincial hospitals call to the institutions of our metropolis to look to their laurels. The circular wards, gay as they looked with Christmas decorations, provoked our admiration, and endorsed our previous approval of this plan of construction. The possible introduction of so much pure air rendered the whole building so fresh, that the "hospital" was well-nigh forgotten. Miss Alice South, the Matron, is to be congratulated on the general appearance and the management of the institution, the internal arrangements of which she conducts. She has lived to see the death of the previous hospital with a veritable Gamp system of nursing, and the birth of the present complete and flourishing institution, which was completed in 1884. The kitchens are at the top of the building, and all the offices are adequate and complete. The out-patients' department is of excellent arrangement. It cannot cause surprise that the hospital continues to advance in popularity, as shown by a steady growth in the number of patients and subscriptions. The present number of beds will soon have to be increased, and extension in this respect is arranged for. The circular wards contain twelve beds in each, and there are besides small wards for obstetric and eye cases. Two endowed beds are memorials to deceased members of the medical staff, which excellent form of recognition might well be initiated in the future both here and elsewhere.

